# Conscious and Non-Conscious Processes Regulate Minority Older Adults’ Sedentary Behavior

**DOI:** 10.1093/geroni/igaa057.1074

**Published:** 2020-12-16

**Authors:** Jaclyn Maher, Derek Hevel, Kourtney Sappenfield, Heidi Scheer, Christine Zecca, Laurie Kennedy-Malone

**Affiliations:** 1 University of North Carolina at Greensboro, Greensboro, North Carolina, United States; 2 UNCG School of Nursing, Greensboro, North Carolina, United States

## Abstract

Accumulating evidence suggests that sedentary behavior (SB), or time spent sitting, is regulated by both conscious (e.g., intentions) and non-conscious (e.g., habits) motivational processes. Much of the work investigating these processes has employed summary-based measures of typical motivation and behavior. This study employed ecological momentary assessment (EMA) methods and accelerometry to determine the extent to which conscious and non-conscious processes regulate minority older adults’ momentary decisions to engage in SB. Over the course of the 8-day study, minority older adults (N=91; age range: 60-89 years, 96% Black/African American) answered 6 EMA questionnaires/day on a mobile phone and wore an ActivPAL activity monitor to measure SB. EMA questionnaires assessed momentary intentions to limit SB over the next two hours. SB habit strength was self-reported at an introductory session. Results from a multilevel linear regression model indicated that on occasions when individuals had stronger intentions than usual to limit SB, they subsequently engaged in less SB (b=-3.72, p<0.01). Individuals who had stronger SB habits, tended to engage in more SB (b=3.00, p<0.01). An additional multilevel model revealed that habits did not significantly moderate the association between momentary intentions and subsequent SB (b=-1.06, p=0.09). In conclusion, minority older adults’ momentary SB appears to be directly influenced by both conscious and non-conscious motivational processes, though the interactive effects are unclear. Interventions to reduce minority older adults’ SB should include content to increase intentions to limit SB (e.g., information on instrumental and affective consequences) and disrupt habitual SB (e.g., action planning).

